# Morphological Features of the Coccyx in the Turkish Population and Interrelationships Among the Parameters: A Computerized Tomography-Based Analysis

**DOI:** 10.7759/cureus.19687

**Published:** 2021-11-18

**Authors:** Bulent Guneri, Gulay Gungor

**Affiliations:** 1 Orthopaedics and Traumatology, Adana City Training Hospital, Adana, TUR; 2 Radiology, Pamukkale University, Denizli, TUR

**Keywords:** spicule, bone morphology, bone spur, caudal vertebra, coccyx, joints, anatomic variation, bone

## Abstract

Introduction

The coccyx is well-known to be a highly variable structure considering its morphology. To our knowledge, the relationship between the coccygeal types and other morphological features has not been studied yet. In addition to the interrelations among morphological parameters, this study investigated the morphology and morphometry of coccyx more extensively in the adult Turkish population using computerized tomography images.

Methods

Five hundred subjects who underwent pelvic computerized tomography were included in this study. In addition to coccyx type and the counts of coccygeal vertebrae and segments, the presence of coccygeal deviation, sacrococcygeal joint (SCJ) fusion, SCJ subluxation, intercoccygeal joint (ICJ) fusion, and coccygeal spicule were evaluated. The coccygeal length, sacrococcygeal angle, and intercoccygeal angle were measured on the digital workstation. The findings were subjected to statistical analyses.

Results

The coccygeal vertebra count ranged between three to five, with an average of 4.04 ± 0.48. The range of coccygeal segment count was between one and five, with an average of 2.53 ± 1.02. ICJ fusion in any segment, SCJ fusion, and SCJ subluxation were identified in 397 subjects (79.4%), 343 subjects (68.6%), and 17 subjects (3.4%), respectively. The coccyx types from the most common to the least common were as follows: type 2, type 1, type 3, type 4, and type 5. Coccygeal deviation to the left side was observed in 71 subjects (14.2%), while coccygeal deviation to the right side was observed in 61 subjects (12.2%). A coccygeal spicule was identified in 73 subjects (14.6%). The subjects’ mean age demonstrated no significant difference considering the ICJ fusion (p=0.271), SCJ subluxation (p=0.51), coccygeal spicule (p=0.337), features of coccygeal deviation (p=0.83), and coccyx types (p=0.11). The subjects with SCJ fusion (50.7 ± 18.3 years) were significantly older than the subjects without SCJ fusion (46.5 ± 18.5 years) (p=0.016). The differences between the coccyx types considering the rate of SCJ fusion (p=0.002), ICJ fusion (p=0.04), and spicule presence (p<0.001) as well as the coccygeal vertebra count (p<0.001) were significant.

Conclusion

The presence of coccygeal spicule, a risk factor for coccydynia, is reported to be 14.6% in this study group that represents the Turkish population. This study indicates an association between the coccyx types and the frequency of SCJ fusion, ICJ fusion, and spicule presence and consequently suggests the significance of the coccyx type among the morphological features to cause susceptibility to coccydynia. Due to the multiplicity of the pain generators in the coccygeal region that is established by previous reports, the comparisons of different human populations and the knowledge on the interrelations between the morphologic parameters might facilitate the comprehension of the etiology of coccydynia. The clarification of interrelationship existence among the coccygeal morphological parameters requires further investigations.

## Introduction

The coccyx, commonly known as the tailbone, is the most caudal part of the vertebral column in humans. This triangular-shaped bone generally consists of three to five vertebrae, interconnected by fibrous joints and ligaments, while the base of the coccyx articulates with the apex of the sacrum through the sacrococcygeal joint (SCJ) [1]. Although the coccyx appears to be rudimentary, it has been shown to function as an attachment site for the ligaments, tendons, and pelvic floor muscles [1,2]. Moreover, the coccyx contributes to weight-bearing in the sitting position and constitutes a three-point support model with two ischial tuberosities [2].

Pain that occurs in the location of the coccyx, also called coccydynia, can be quite disturbing, particularly during sitting. The causes of coccydynia include trauma, inflammation, tumor, and disc degeneration [2-5]. The association between the coccyx morphology and coccydynia has been defined as well [4]. The studies on the variations of coccygeal morphology among different populations, including the Turkish population, are present in the literature [4,6-12]. To our knowledge, the frequency of coccygeal spicule presence and the relation of age with the fusion of SCJ and intercoccygeal joint (ICJ) in the Turkish population as well as the relation of the coccygeal types with other morphological features (more specifically, coccygeal spicule and the fusion of the joints) have not been studied yet. The present study investigated the morphology and morphometry of coccyx more extensively in the adult Turkish population using computerized tomography (CT) images. Furthermore, the interrelations between the morphological parameters were examined, and the findings of the present study were compared with the findings of previous reports on the Turkish population and other populations.

## Materials and methods

Study design

A retrospective review was performed on consecutive patients who underwent pelvic CT examination between April 2018 and October 2018 in the authors’ institution. The exclusion criteria were the history of previous coccygeal or sacral injury and the presence of malformation in the coccygeal vertebrae and/or sacral vertebrae due to congenital disorders, infection, or space-occupying lesions. The institutional review board of Kahramanmaras Sutcu Imam University approved the study protocol (approval date: April 4, 2018; issue: 7-10). 

Computerized tomography protocol and measurements

The CT images were obtained using a 640 multislice CT scanner (Aquilion ONE, Toshiba Medical Systems, Otawara, Japan). The images of the patients were assessed with axial scans and sagittal and coronal reformats. The scan parameters were (120 kVp, variable mA and sec.), reconstruction thickness 1.5 mm, noise index 23, pitch 0.984:1, and gantry rotation time 0.27 s. The measurements were performed on Workstation (Apple Inc., Cupertino, California, US; OsiriX V.4.9 imaging software Pixmeo, Switzerland). All CT images were taken from the patients lying supine during the imaging procedures. The radiologic evaluation was performed by the author experienced in musculoskeletal system radiology (GG).

**Figure 1:**
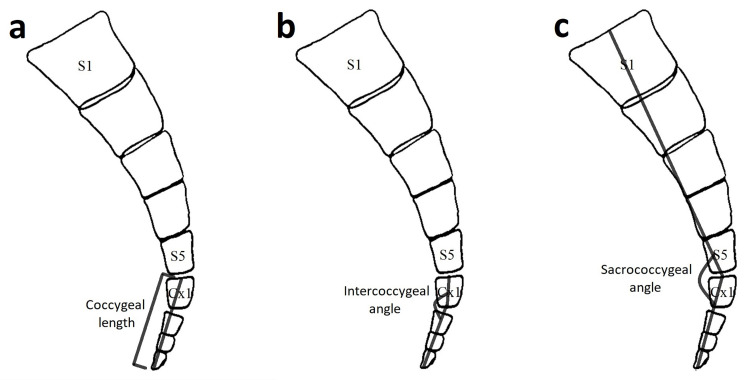
The schematic representation of the measurements related to the coccygeal morphometry a. Coccygeal length, b. Intercoccygeal angle, c. Sacrococcygeal angle.

The CT images were analyzed in terms of coccygeal morphology and morphometry. The evaluation of coccygeal morphology included the identification of coccyx type (based on modified Postacchini and Massobrio classification), the numbers of coccygeal vertebrae and coccygeal segments, the presence of coccygeal deviation to the right or left side as well as the presence of SCJ fusion, SCJ subluxation, ICJ fusion, and coccygeal spicule (Table 1). The evaluation of coccygeal morphometry included the measurement of coccygeal length, sacrococcygeal angle, and intercoccygeal angle (Figure 1) [4,12].

**Table 1:** The definitions of the specific morphologic and morphometric parameters

Morphologic parameters	Definition
Coccyx type (based on the modified Postacchini and Massobrio classification) [4,12]	Type 1: the coccyx is slightly curved and its tip points downwards; type 2: the coccyx is significantly curved and its tip points forwards; type 3: the coccyx is sharply angulated at the intercoccygeal joint; type 4: the coccyx has subluxation at the sacrococcygeal joint or first intercoccygeal joint; type 5: retroverted coccyx.
Number of coccygeal segment(s) [4]	The calculation is based on the principle considering the independent (unfused) coccygeal vertebrae and accepts the fused vertebrae as one segment.
Sacrococcygeal/intercoccygeal joint fusion [12]	Continuity between the bones at the sacrococcygeal/intercoccygeal joint.
Coccygeal spicule	Bony projection originating from the most caudal aspect of the coccyx.
Morphometric parameters	Definition
Coccygeal length	The distance between the midpoint of the upper endplate of first coccygeal vertebra and the tip of the coccyx, as depicted in Figure 1a.
Intercoccygeal angle	The angle is formed between the lines passing through the middle of first coccygeal vertebra and the middle of the rest of coccygeal vertebrae in the mid-sagittal plane, as depicted in Figure 1b.
Sacrococcygeal angle	The angle is formed by the intersection of a line between the midpoint of the upper endplate of first sacral vertebra and the midpoint of the upper endplate of first coccygeal vertebra and a line between the midpoint of the upper endplate of first coccygeal vertebra and the tip of the coccyx, as depicted in Figure 1c.

Statistical analysis

The statistical analysis was performed using IBM SPSS version 20.0 for Windows (IBM Corp., Armonk, NY, USA). Continuous variables were presented as mean ± standard deviation, and categorical variables were presented as frequency distribution and percentage. Normality testing was performed using the Kolmogorov-Smirnov test. A comparative analysis between two or more independent groups was performed using the Mann-Whitney U test, the student's t-test, and analysis of variance (ANOVA) for continuous variables. The Chi-square test was used for the comparison of categorical data. A p-value of less than 0.05 was considered statistically significant.

## Results

Five hundred subjects, consisting of 259 (51.8%) females and 241 (48.2%) males, were included and analyzed. The female subjects were slightly younger than the male subjects (48.2 ± 19.2 years vs. 50.6 ± 17.5 years, respectively, p=0.125).

Coccygeal morphology

The number of the coccygeal vertebrae in total and the gender-specific distribution are demonstrated in Table 2. The number of the coccygeal vertebrae ranged between three to five, with an average of 4.04 ± 0.48. The mean number of the coccygeal vertebrae was significantly low (3.98 ± 0.51) in the female subjects as compared to the male subjects (4.1 ± 0.45) (p=0.004). ICJ fusion in any segment was identified in 397 subjects (79.4%), while no ICJ fusion was observed in 103 subjects (20.6%), and the gender-specific distribution demonstrated no significant difference (p=0.558). The number of the coccygeal segments ranged from one to five in the male and female subjects. The mean number of the coccygeal segments was 2.47 ± 1.06 in the females, 2.59 ± 0.98 in the males, and 2.53 ± 1.02 in total. The gender-specific difference as per the numbers of the coccygeal segments was not significant as well (p=0.263). The coccygeal vertebrae were completely ossified in 73 subjects (14.6%); 12 subjects (2.4%) had three, 56 subjects (11.2%) had four, and five subjects (1%) had five fused vertebrae. Out of 73 subjects with complete coccygeal fusion, 45 females (61.6%) compared to 28 males (38.4%), 66 subjects (90.4%) had associated SCJ fusion.

**Table 2:** The overall and gender-specific distribution of the numbers of the coccygeal vertebrae

The numbers of coccygeal vertebrae	n (percentage) in females	n (percentage) in males	n (percentage) in total
Three	36 (7.2%)	13 (2.6%)	49 (9.8%)
Four	193 (38.6%)	191 (38.2%)	384 (76.8%)
Five	30 (6.0%)	37 (7.4%)	67 (13.4%)
Overall (3-5)	259 (51.8%)	241 (48.2%)	500 (100%)

The SCJ fusion was identified in 343 subjects (68.6%), while no SCJ fusion was observed in 157 subjects (31.4%). The gender-based difference considering the frequency of SCJ fusion was minor (p=0.479). The SCJ subluxation was observed in 17 subjects (3.4%), while no subluxation was observed in 483 subjects (96.6%). The distribution of genders considering the SCJ subluxation demonstrated no significant difference as well (p=0.279). 

**Figure 2:**
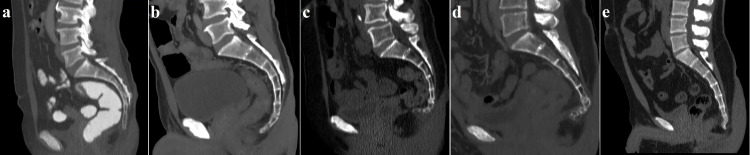
Examples of computerized tomography images in the sagittal plane showing five types of coccyx a. Type 1 coccyx in a 61-year-old female participant. b. Type 2 coccyx in a 54-year-old male participant. c. Type 3 coccyx in a 69-year-old female participant. d. Type 4 coccyx in a 53-year-old female participant. e. Type 5 coccyx in a 24-year-old male participant.

**Figure 3:**
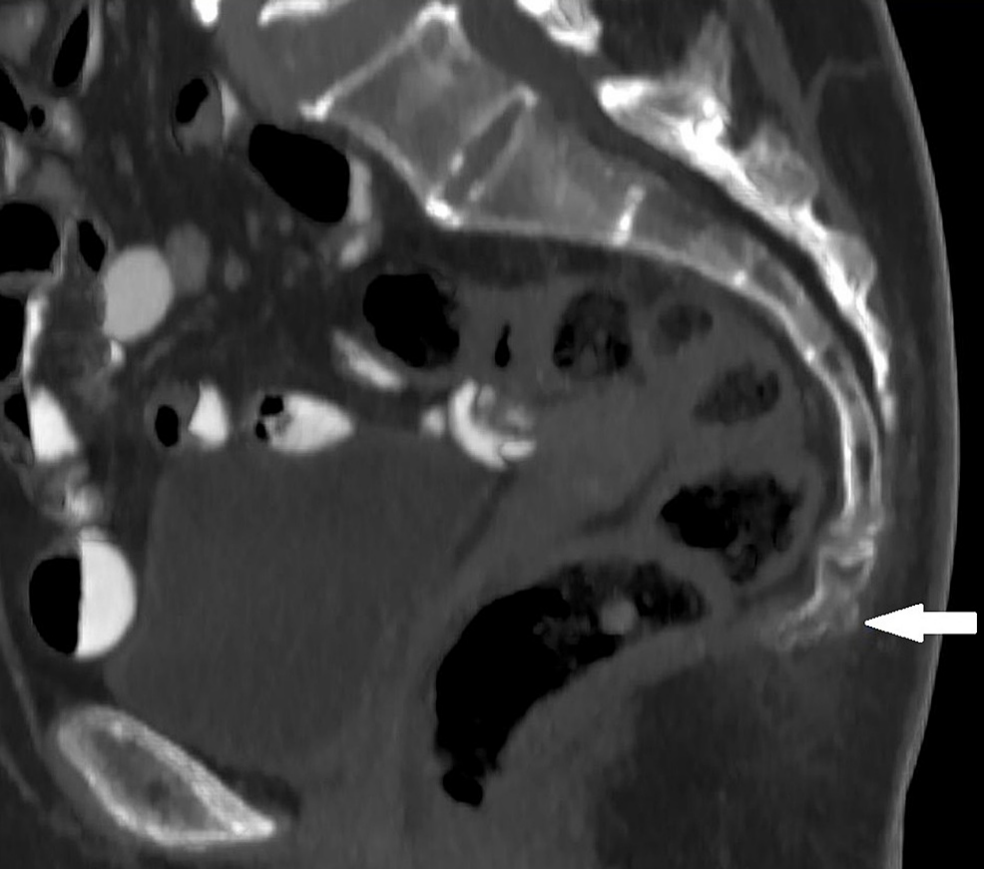
Example of computerized tomography image in the sagittal plane showing a coccygeal spicule (white arrow).

The ranking of the coccyx types from the most common to the least common was as follows: type 2, type 1, type 3, type 4, and type 5 (Figure 2). The distribution of the coccyx types among genders was similar (p=0.631) (Table 3). Coccygeal deviation to the left side was observed in 71 subjects (40 females - 8.0%; 31 males - 6.2%; 14.2% overall), while coccygeal deviation to the right side was observed in 61 subjects (26 females - 5.2%; 35 males - 7.0%; 12.2% overall). A coccyx without deviation to either side was identified in 368 subjects (193 females - 38.6%; 175 males - 35.0%; 73.6% overall). The comparison of genders as per the features of coccygeal deviation did not demonstrate a significant difference (p=0.259). A coccygeal spicule was identified in 73 subjects (14.6%); 39 of them (53.4%) were female and 34 (46.6%) were male, indicating no significant gender-based difference (p=0.764) (Figure 3).

**Table 3:** The gender-specific distribution of the coccyx types

Coccyx types	Female – n (%)	Male – n (%)	Total – n (%)
Type 1	38 (7.6%)	25 (5.0%)	63 (12.6%)
Type 2	174 (34.8)	174 (34.8%)	348 (69.6%)
Type 3	28 (5.6%)	27 (5.4%)	55 (11.0%)
Type 4	17 (3.4%)	13 (2.6%)	30 (6.0%)
Type 5	2 (0.4%)	2 (0.4%)	4 (0.8%)
Overall	259 (51.8%)	241 (48.2%)	500 (100%)

The mean age of the subjects did not demonstrate a significant difference considering the ICJ fusion presence (p=0.271), SCJ subluxation presence (p=0.51), spicule presence (p=0.337), and the features of coccygeal deviation (p=0.83) as well as the coccyx types (p=0.11). On the other hand, the mean age of the subjects with SCJ fusion (50.7 ± 18.3 years) was significantly high as compared to the mean age of the subjects without SCJ fusion (46.5 ± 18.5 years) (p=0.016).

The differences between the coccyx types considering the rate of SCJ fusion (p=0.002), ICJ fusion (p=0.04), and spicule presence (p<0.001) as well as the numbers of coccygeal vertebrae (p<0.001) were significant (Table 4). However, the difference among the coccyx types as per the features of coccygeal deviation was minor (p=0.115).

**Table 4:** The relationship of coccyx types with the presence of SCJ fusion, ICJ fusion, and coccygeal spicule and the mean number of the coccygeal vertebrae * Chi-square; ** ANOVA. ICJ: intercoccygeal joint, SCJ: sacrococcygeal joint, SD: standard deviation.

	Type 1	Type 2	Type 3	Type 4	Type 5
SCJ fusion (-): n (%)	8 (1.6%)	113 (22.6%)	21 (4.2%)	12 (2.4%)	3 (0.6%)
SCJ fusion (+): n (%)	55 (11.0%)	235 (47.0%)	34 (6.8%)	18 (3.6%)	1 (0.2%)
	p=0.002*				
ICJ fusion (-): n (%)	7 (1.4%)	71 (14.2%)	14 (2.8%)	11 (2.2%)	0
ICJ fusion (+): n (%)	56 (11.2%)	277 (55.4%)	41 (8.2%)	19 (3.8%)	4 (0.8%)
	p=0.04*				
Coccygeal spicule (-): n (%)	43 (8.6%)	310 (62.0%)	47 (9.4%)	27 (5.4%)	0
Coccygeal spicule (+): n (%)	20 (4.0%)	38 (7.6%)	8 (1.6%)	3 (0.6%)	4 (0.8%)
	p<0.001*				
The numbers of coccygeal vertebrae: mean±SD	3.82±0.49^b,c^	4.04±0.44^a,c^	4.25±0.55^a,b,e^	4.03±0.49	3.50±0.57^c^
	p<0.001**				

Coccygeal morphometry

The mean length of the coccyges was 38.32 ± 8.18 mm. The coccyges were significantly longer in the male subjects (mean length: 40.15 ± 7.51 mm) as compared to the female subjects (mean length: 36.62 ± 8.41 mm) (p<0.001). According to the mean values, the type 1 coccyx was the longest (mean length: 38.73 ± 8.18 mm), whereas the type 5 coccyx was the shortest (mean length: 34.45 ± 9.49 mm). However, the comparison of the coccygeal types considering the lengths demonstrated no significant difference (p=0.268).

The mean sacrococcygeal angle was 111.39 ± 13.51 degrees. The mean sacrococcygeal angle of the females (109.94 ± 14.01 degrees) was significantly lower than the mean sacrococcygeal angle of the males (112.95 ± 12.8 degrees) (p=0.006). The overall mean intercoccygeal angle was 140.27 ± 18.18 degrees. The mean intercoccygeal angle was 140.39 ± 19.68 degrees in the females and 140.14 ± 16.44 degrees in the males, demonstrating no significant gender-based difference (p=0.836).

## Discussion

The coccyx has been reported to be a highly variable structure considering its morphology, including the number of coccygeal vertebrae and segments [1]. The presence of four vertebrae was the most common finding as per the coccygeal vertebra count in this study, followed by the presence of five and three vertebrae in descending order of frequency. Karayol et al. reported an identical ranking for the coccygeal vertebra count in their MRI-based research on the Turkish population [8]. On the other hand, Hekimoglu and Ergun, who have investigated the coccygeal morphology of the Turkish population using CT scan, reported the order from the most frequent number to the least as follows: four, three, five, and two and one [6]. A single study previously notified the frequency of one-segment coccyx (i.e., the fusion rate of entire coccygeal vertebrae) in the Turkish population, and the reported rate was 2.8% [11]. One segment coccyx was remarkably more frequent in the present study, i.e., 14.6%. The discordant results of these two studies may arise from the diversity of study groups’ constitutions as well as the criteria applied to evaluate ICJ fusion.

SCJ fusion was identified in 68.6% of the present study group. While Hekimoglu and Ergun notified SCJ fusion in 152 (67.8%) out of 224 subjects [6], Tetiker et al., who have studied coccygeal morphology of the Turkish population using MRI, reported the fusion rate as 23.8% in the male subjects and 21.6% in the female subjects [11]. SCJ subluxation, on the other hand, was uncommon (3.4%) in this study and inconsistent with the finding of Karayol et al., who only studied the rate of SCJ subluxation (27.3%) in the Turkish population previously [8]. In addition to some degree of diversity in the criteria to evaluate these morphological features, the evident dissimilarity between those reported rates seems to indicate variability in SCJ fusion and subluxation rates among the human populations.

The relationship between the frequency of SCJ fusion and age seems controversial. A study on the Korean population notified no association between the age of the participants and the frequency of SCJ fusion [10]. A multicenter study including participants from New Zealand and France also reported no relationship between age and the fusion rate of SCJ and ICJ [12]. On the contrary, another study including Korean participants reported significantly higher fusion rates of SCJ in the eighth, ninth, and tenth decades [4]. The relationship between age and the fusion rate of SCJ and ICJ has not been reported in the Turkish population to date. The findings of the present study indicate significantly high age in the participants with SCJ fusion as compared to the participants without fusion, while no significant difference was identified between the ICJ fusion and non-fusion groups. These results suggest fusion of SCJ with aging, presumably in a fraction of individuals.

Coccygeal spicule, a possible cause of coccydynia, may present with resistant coccygeal pain following conservative treatment, and coccygectomy may even be required to relieve the disabling symptoms seen in those patients [13]. Based on the literature search, the prevalence of coccygeal spicule in the Turkish population has not been reported to date. A spicule was identified in 14.6% of the subjects included in this study. A study on the Indian population reported spicule presence in 18 (8.4%) out of 213 subjects [7], while the multicenter study reported the existence of coccygeal spicule in 23% of the study population [12]. The calculated rate of the present study stands within the range of the frequencies mentioned above.

The presence or absence of coccygeal deviation in the coronal plane is an additional morphological feature assessed in the present study. Only one prior study, conducted by Hekimoglu and Ergun, included the assessment of coccygeal deviation in the Turkish population [6]. The deviation was reported to be identified in 17 out of 224 subjects, with 10 deviations to the left side. The rate of coccygeal deviation to either side was more frequent (14.2%), with a major predominance of the deviation to the right side (61 out of 71 coccygeal deviations) in the present study, suggesting variability in terms of coccygeal deviation when the findings of these two studies are compared.

The Turkish population has previously been investigated considering the coccygeal types as well [6,8,9,11]. The study, which was based on CT images and the classification ofPostacchini and Massobrio,evaluated the coccyx types in asymptomatic individuals [9,14]. The authors reported type 1, defined as a slightly forward-curved coccyx, to be the most common type in their study involving 92 subjects. Type 4, representing the subluxation of SCJ or ICJ, was mentioned to be non-existent in the study group. Besides, they notified two patients with retroverted coccyges (i.e., type 5 according to modified Postacchini and Massobrio classification) that were not defined by the classification used. Hekimoglu and Ergun mentioned type 1 as the most common type (60.7%) type 5 as the least common type (0.9%), while type 4 was reported to be non-existent in the study group [6]. Karayol et al. reported the coccygeal types in descending order of frequency as follows: type 2, type 1, type 3, type 4 and 5 [8]. The results of the latter study and the present study apparently show concordance, which may be attributed to the significant geographical proximity of the study populations as compared to the other two studies and the conceivable similarities between these two study groups considering hereditary and/or environmental factors. On the other hand, the influence of hereditary and environmental factors on the coccyx types is currently unestablished and might be a subject of further investigation.

The gender-based comparison of the coccygeal morphology and morphometry indicated significant differences regarding the coccygeal length, coccygeal vertebra count, and sacrococcygeal angle in this study. Significant inter-gender difference related to vertebral counts of the present study contradicts various investigations, including study groups representing diverse human populations [4,11,12]. Karayol et al., on the other hand, reported significantly higher vertebral count in the male subjects, demonstrating consistency with the present study [8]. A study on the Indian population and the only study, which investigated the difference between genders considering sacrococcygeal angle in the Turkish population, have reported a significantly high mean value of the sacrococcygeal angle in the male participants compared to the female participants [7,11], similar to the present study. However, two previous studies on the Korean population and a multicenter study have indicated insignificant inter-gender differences [4,10,12]. Therefore, the gender-based difference in the coccygeal morphology and morphometry seems controversial.

A distinctive feature of this study is the comparison of coccyx types as per the morphological parameters. Nonetheless, Karayol et al. previously compared the coccyx types only regarding the numbers of coccygeal vertebrae as a morphological feature and reported a significant difference between the types [8]. The analysis of the present study indicated significant differences among the coccygeal types as per the numbers of the coccygeal vertebrae as well as the presence of SCJ fusion, ICJ fusion, and spicule. In the present study, the type 3 coccyx contained the highest vertebrae count with a significant difference compared to type 1, type 2, type 5. The type 5 coccyx contained the lowest vertebrae count with a significant difference compared to type 3. The ratio of the fusion frequency to the non-fusion frequency in SCJ and ICJ conspicuously decreased from type 1 to type 5 (except for the ratio related to ICJ in type 5) as follows: SCJ: 6.87 in type 1, 2.07 in type 2, 1.61 in type 3, 1.5 in type 4, and 0.33 in type 5; ICJ: 8.0 in type 1, 3.9 in type 2, 2.92 in type 3, 1.72 in type 4, 4/0 in type 5. When the least frequently identified coccygeal type in this study, type 5 (n=4), was excluded, coccygeal spicule was proportionally the most and least frequent in the type 1 coccyx and type 4 coccyx, respectively. A retrospective case-control study reported the presence of spicule, type 2 coccyx, and non-fusion at the SCJ as risk factors for idiopathic coccydynia [5]. Indicating an association between the coccyx types and the frequency of spicule presence as well as the frequency of SCJ and ICJ fusion, the present study suggests the significance of the coccyx type among the morphological features that appears to cause susceptibility to coccydynia.

The strengths of the present study are the comparatively large size of the study group and the assessment of interrelation between the coccyx types and other morphological features. The limitations are the assessment by a single observer and the absence of the participants’ stature measurements.

## Conclusions

The presence of coccygeal spicule, a risk factor for coccydynia, is reported to be 14.6% in this study group that represents the Turkish population. Significantly high age in the subjects with SCJ fusion compared to the subjects without fusion appears to support the hypothesis of SCJ fusion process with advancing age. In addition to significant gender-based differences considering the coccygeal length, coccygeal vertebra count, and sacrococcygeal angle, this study indicates association between the coccyx types and the frequency of SCJ fusion, ICJ fusion, and spicule presence. Consequently, the significance of the coccyx type among these morphological features, which are reported to cause susceptibility to coccydynia, could be suggested. Due to the multiplicity of the pain generators in the coccygeal region that is established by previous reports, the comparisons of different human populations and the knowledge on the interrelations between the morphologic parameters might facilitate the comprehension of the etiology of coccydynia. The clarification of the existence of interrelationship among the coccygeal morphological parameters, initially suggested by this study, requires further investigations.
